# Supplementary material for: Learning curve and surgical outcome of robotic assisted colorectal surgery with ERAS program
Source: Sci Rep. 2022 Nov 29;12:20566. doi: 10.1038/s41598-022-24665-w (PMC9709162; doi:10.1038/s41598-022-24665-w)
Supplement: Supplementary file 1 — Supplementary Information 1. [file 41598_2022_24665_MOESM1_ESM.zip › LSC Raw data-Ñ[▒K/085/O_ñw╝╨░O▒Kñσ.pdf]

正式摘要(電子簽章：000000195282625)

W55 / W55

臺中榮民總醫院

Operation Note

[redacted]

- 手術日期：2020/07/22
- 手術醫師：VS 林俊余，林昌霖，R 蔡濬樟
- Anesthesia : GE
- Position : Lithotomy
- Aseptic agents : Betadine Alcohol , Betadine Aqua

Diagnosis

- Pre-operative diagnosis : Malignant tumor of distal D-colon with total obstruction, s/p loop T-colostomy
- Post-operative diagnosis : Malignant tumor of distal D-colon with total obstruction, s/p loop T-colostomy, s/p 3D laparoscopic left hemicolectomy on 20200722, cT4aN2bM0.

Surgical Indication

- For curative operation

Operation

- 1. 3D Laparoscopic left hemicolectomy with side-to-side anastomosis, end-GIA 60/4.1*3, isoperstalsis, re-inforcement with silk: done.
  2. Vessel ligation: left branch of middle colic and left colic vessels, S1 vessel.
  3. Cut margin: proximal 9 cm, distal 8 cm.
  4. Reperitonealization: Nil.
  5. Drain : nil.

Finding

- 1. One 10*5*4 cm Borrmann's type III ulcerative tumor at distal D-colon, which invaded through the colon serosa, but didn't involved the adjacent organs or structures. Clinical stage: T4aN2bM0.
  2. There were servel(>6) enlarged indurated lymph nodes at mesocolon and left branch of middle colic vessels.
  3. There was no visible liver metastasis, no visible peritoneal seeding

Procedure

- General endotracheal anesthesia was induced and then the patient was placed in supine position. Preoperative antibiotics were given. A Foley catheter and nasogastric tube were placed. The abdomen and perineum was prepped and draped in standard sterile fashion. A small vertical incision was made below the umbilicus and a 12mm trochar was inserted into the peritoneal cavity by open method. Peumoperitoneum was established with CO2 inflation. Laparoscopy was inserted and then a 12mm trochars were inserted over RLQ of abdomen. three 5mm trochars were inserted over RUQ, LUQ and LLQ of abdomen. The liver, omentum and peritoneum were inspected for evidence of metastatic disease. No metastatic disease was noted. Enterolysis was performed for free of omentum and previous abdominal incision wound. A mass lesion was noted in the splenic flexure. The small bowel was inspected and retracted to upward by grasper. After identification of IMV, the IMV was held by grasper and the peritoneum overlying the mesentery was then scored with electrocautery. Dissection was done above the Gerota's fascia and toward the splenic flexure. The left branch of middle colic artery and left colic artery were then identified, ligated with endoclips, and transected. Using electrocautery, omentum was freed from the transverse colon. The colon was then freed from its peritoneal attachments along the line of Toldt proximally from the splenic flexure and

- distally to the pelvic inlet. Splenic flexure and descending colon were mobilized. A small midline incision was made. The descending colon was delivered into the incision and points of transection were selected proximally and distally. The bowel was divided and then the specimen was removed. The two ends of the bowel were checked and found to be viable, with excellent blood supply. The fat was gently cleared from the terminal 2-3 mm of the bowel ends. The two end of the bowel were brought into apposition and found to lie comfortably without excessive tension. Side-to-side anastomosis was performed with endo-GIA 60/4.1. Interrupted 3-0 silk Lambert sutures were used to re-inforcement at outer layer. The anastomosis was checked and found to be intact and widely patent. The fascia of lower midline wound was closed with interrupted sutures of No.1 Vicryl. The fascia of the two 12 mm trochar wound was closed with 1-0 Dexon. the skin was closed by 3-O nylon interrupt suture. The patient tolerated the procedure well and was brought to the post-anesthesia care unit in stable condition. / CR Lin, Chang-Lin
- Estimated blood loss: minimum
- 器械數量: 正確
- 紗布數量: 正確

Image

- PAINT
  - colon & rectum

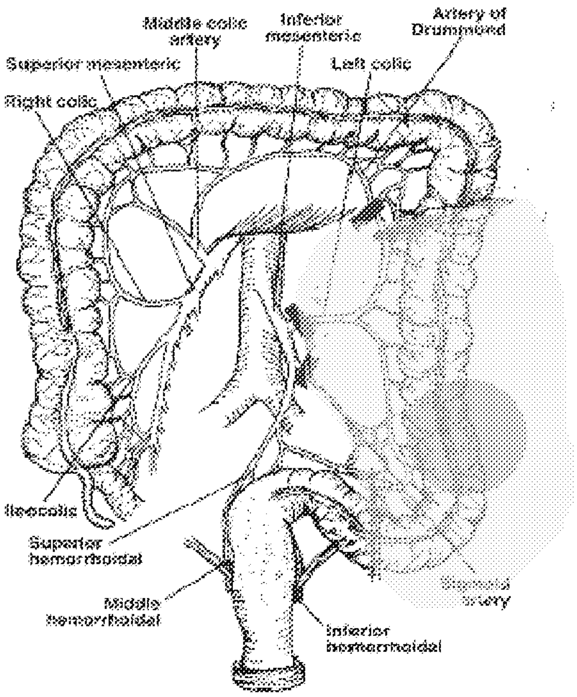

  - abdomen

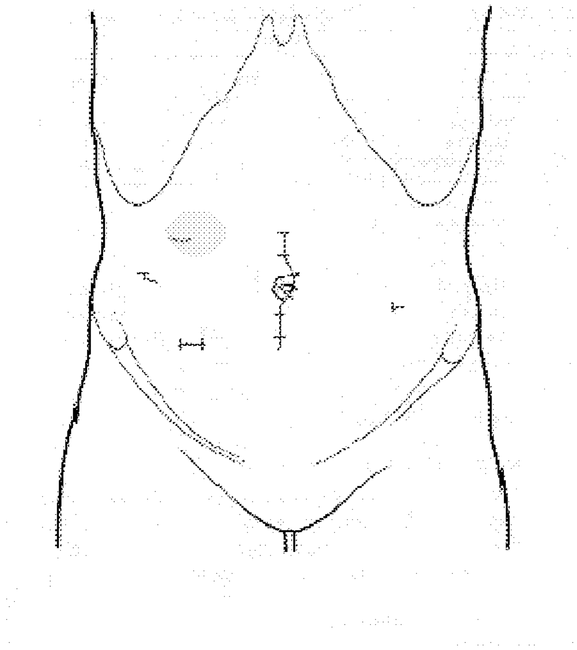

編輯主治 2222H林俊余、主治 2320B林昌霖

正式摘要(電子簽章：000000195282625)

W55 / W55

doctorRank:B
